# The Triglyceride-Glucose-Chinese Visceral Adiposity Index (TyG-CVAI) outperforms other insulin resistance indices in its association with cardiovascular-kidney-metabolic syndrome severity in type 2 diabetes: a comparative study

**DOI:** 10.3389/fnut.2026.1817071

**Published:** 2026-05-08

**Authors:** Jian Yang, Zhiling Deng, Bingsong Xie, Feng Tian, Youxu Leng, Hairong Zhou

**Affiliations:** 1 Department of General Medicine, Longhua District People's Hospital, Shenzhen, China; 2 Department of General Medicine, The Eighth Affiliated Hospital of Sun Yat-sen University, Shenzhen, China; 3 Department of Obstetrics and Gynecology, Longhua District Central Hospital, Shenzhen, China

**Keywords:** cardiovascular-kidney-metabolic syndrome, community-based study, insulin resistance, TyG-CVAI, type 2 diabetes

## Abstract

**Background:**

The Cardiovascular-Kidney-Metabolic (CKM) syndrome integrates the pathophysiology of metabolic disease, chronic kidney disease, and cardiovascular disease. Accessible biomarkers for risk stratification are urgently needed, especially in high-risk populations like patients with type 2 diabetes (T2D). We compared the novel Triglyceride-Glucose-Chinese Visceral Adiposity Index (TyG-CVAI) against established insulin resistance (IR) surrogates for its association with CKM severity in T2D.

**Methods:**

This cross-sectional study analyzed 5,609 adults with T2D from community health centers in Shenzhen, China (2023). CKM stages (2–4) were defined per AHA criteria. The associations of TyG, TyG-BMI, TyG-WHtR, and TyG-CVAI with CKM stage were assessed using Spearman correlation and multivariable ordinal logistic regression. Discriminatory performance for late-stage CKM (Stages 3–4) was evaluated using ROC curve analysis.

**Results:**

TyG-CVAI showed the strongest correlation with CKM stage (ρ = 0.326, *P* < 0.001). In fully adjusted models, a one-SD increase in TyG-CVAI was associated with higher odds of advanced CKM stage (OR = 1.20, 95% CI: 1.11–1.29, *P* < 0.001). This association was consistent across sex and hypertension subgroups but stronger in participants aged ≥60 years (*P* for interaction = 0.036). For discriminating late-stage CKM, TyG-CVAI achieved a significantly higher AUC (0.720, 95% CI: 0.705–0.734) than TyG (0.574), TyG-BMI (0.570), and TyG-WHtR (0.587) (all *P* < 0.001).

**Conclusion:**

In adults with T2D, TyG-CVAI demonstrates a superior association with CKM severity and better diagnostic accuracy for late-stage CKM compared to simpler TyG-based indices. It represents a promising, accessible biomarker for identifying higher CKM stage severity, with better discriminatory ability than simpler TyG-based indices, in this high-risk population.

## 1 Introduction

The Cardiovascular-Kidney-Metabolic (CKM) syndrome delineates a pathophysiological convergence in which metabolic dysfunction, chronic kidney disease (CKD), and cardiovascular disease (CVD) interact synergistically, leading to substantial morbidity and mortality. The American Heart Association (AHA) has recently formalized CKM as a staged continuum, emphasizing prevention and early risk stratification from excess adiposity to overt CVD, and underscoring the critical need for accessible, stage-specific biomarkers to guide interventions (1, 2). The public health burden of CKM is considerable, as metabolic disorders and CKD potentiate cardiovascular risk, with their overlap accounting for a major share of the clinical burden attributed to this syndrome (3).

Within the CKM continuum, type 2 diabetes (T2D) represents a pivotal and aggravating factor. It not only propels the transition through successive CKM stages but also compounds the susceptibility to cardiac and renal endpoints. Consequently, individuals with diabetes constitute a critical target for preventive strategies and refined risk assessment within the CKM paradigm (4). Globally, the population with diabetes exceeds 500 million adults, highlighting the magnitude of this high-risk subgroup (5). In patients with established T2D, chronic hyperglycemia, advanced glycation end-products, and persistent insulin resistance create a unique metabolic milieu that accelerates endothelial dysfunction, renal microvascular injury, and atherogenesis—pathways that are less pronounced or only emerging in general populations without diabetes (6–8). Thus, T2D represents not merely a risk factor but a distinct pathophysiological state within the CKM continuum (9). Insulin resistance (IR) serves as a fundamental mechanistic link connecting adiposity, dyslipidemia, hyperglycemia, and end-organ damage in CKM progression. Accordingly, surrogate measures of IR have been extensively explored as practical risk indicators in both population and clinical settings (10, 11).

Among these surrogates, the triglyceride-glucose (TyG) index—calculated from fasting triglycerides and glucose—has gained recognition as a simple yet validated proxy for IR. It has been consistently associated with incident diabetes, CVD, and CKD across diverse cohorts (12–14). To enhance predictive capability beyond the TyG index alone, composite indices incorporating anthropometric or adiposity measures (e.g., TyG-BMI, TyG-WC, TyG-WHtR) have been developed. Several studies report their incremental value in associating with cardiovascular and cerebrovascular outcomes (15–17). Recent analyses of national cohorts like CHARLS have further shown that TyG-derived composites correlate with incident CVD, stroke, and cardiometabolic multimorbidity across CKM stages 0–3, supporting their potential for cross-sectional risk stratification (18, 19).

The triglyceride-glucose-Chinese visceral adiposity index (TyG-CVAI) is a novel composite that integrates the metabolic signal of the TyG index with the Chinese Visceral Adiposity Index (CVAI), a validated estimator of visceral fat in Chinese populations (20). By combining parameters reflecting triglycerides, fasting glucose, waist circumference, and age, TyG-CVAI is designed to concurrently capture dyslipidemia, glycemic status, visceral adiposity burden, and age-related risk accumulation—key pathways in CKM progression. Preliminary evidence from CKM cohorts suggests TyG-CVAI and similar visceral-adiposity-weighted TyG composites may surpass simpler indices in associating with stroke and other cardiovascular events, though available data remain limited and inconsistent (18, 21).

Notwithstanding these advances, key knowledge gaps persist. Notably, a direct and comprehensive benchmarking of TyG-CVAI against a spectrum of conventional IR surrogates is lacking, particularly within cohorts confined to established type 2 diabetes—the subgroup inherently at the greatest risk within the CKM spectrum. Furthermore, the consistency of TyG-CVAI's utility across relevant clinical subgroups (e.g., defined by age, sex, or hypertension status) and its added value beyond traditional risk factors are not fully characterized. Much of the existing evidence originates from general or mixed-risk populations, which may limit its direct applicability to diabetes management pathways where tailored screening thresholds are needed.

To address these evidence gaps, we undertook a head-to-head comparative analysis using a real-world, community-based cohort of patients with T2D from Shenzhen, China (*n* = 5,609). This study was designed with three primary objectives: first, to assess and compare the associations of multiple IR indices—TyG, TyG-BMI, TyG-WHtR, and TyG-CVAI—with advancing CKM stages; second, to quantify the discriminatory performance of TyG-CVAI relative to the other indices for identifying late-stage CKM; and third, to examine potential effect modification by key demographic and clinical factors.

## 2 Materials and methods

### 2.1 Study design and population

This retrospective, cross-sectional analysis utilized de-identified electronic health records (EHRs) from 45 community health centers in Shenzhen, China, covering the calendar year 2023. The initial search identified 6,800 adults (age ≥18 years) with a documented diagnosis of type 2 diabetes mellitus (T2DM). Exclusion criteria were applied sequentially: 650 participants lacked data required to calculate any of the studied insulin resistance (IR) indices; 480 had insufficient information to assign a CKM stage; and 61 presented extreme or implausible values in key variables (e.g., BMI < 15 or >50 kg/m^2^, triglycerides >20 mmol/L). Extreme BMI values were excluded to minimize the influence of severe malnutrition or extreme obesity, which are rare in community-based T2D populations and may bias associations. Triglyceride levels >20 mmol/L were excluded as such extreme hypertriglyceridemia is often due to genetic dyslipidemias rather than typical T2D-related insulin resistance, and may represent outliers that disproportionately influence regression estimates. The final analytic cohort comprised 5,609 participants. The study protocol was reviewed and approved by the Ethics Committee of Longhua District People's Hospital (Approval No. 2026.03; Approval Date: January 2026). The requirement for written informed consent was waived by the committee due to the retrospective nature of the study, the use of fully de-identified data, and the absence of any direct contact with participants. All data were used exclusively for this study, stored in a controlled environment, and handled in accordance with the principles of the Declaration of Helsinki.

### 2.2 Definitions of CKM syndrome stages

CKM stage for each participant was determined based on data from the index visit in 2023, following the AHA Presidential Advisory on Cardiovascular-Kidney-Metabolic Health, with pragmatic adaptations for EHR-based variables (1).

Stage 2 (Metabolic risk factors and/or CKD): all participants had T2DM, fulfilling the core criterion for Stage 2. The presence of additional metabolic risk factors (e.g., hypertension) or moderate-to-high risk CKD (KDIGO categories G2-G3 based on eGFR and albuminuria) was recorded but did not alter this baseline stage assignment.

Stage 3 (Subclinical CVD or high predicted risk): participants were classified as Stage 3 if they met any pre-specified criterion indicating elevated cardiovascular risk in the absence of clinically diagnosed CVD: (a) a high predicted 10-year CVD risk (Framingham Risk Score ≥10%); the Framingham Risk Score was calculated using the standard 10-year hard CVD risk equation (myocardial infarction and coronary death) incorporating the following variables: age, gender, total cholesterol, HDL cholesterol, systolic blood pressure, smoking status, and diabetes status (22). We acknowledge that the original Framingham equation was derived from a White American cohort; no race-specific version for Chinese populations is universally accepted, so the standard equation was used as a pragmatic risk stratification tool. or (b) very high-risk CKD (KDIGO “very high-risk” category or CKD G4-G5) (23).

Stage 4 (Clinical CVD): this stage was assigned to participants with a documented history of clinical cardiovascular disease, including coronary heart disease, heart failure, stroke, peripheral artery disease, or atrial fibrillation, as identified via diagnostic codes or physician records in the EHR.

For analytical purposes, CKM stage was treated as an ordered categorical variable (Stage 2 → 3 → 4). A binary outcome termed “late-stage CKM” was defined as the presence of Stage 3 or 4 vs. Stage 2 alone.

### 2.3 Calculation of insulin resistance indices

Four key insulin resistance indices were calculated from anthropometric and laboratory data obtained at the index visit:

Triglyceride-glucose index (TyG): Ln[fasting triglycerides (TG, mmol/L) × fasting plasma glucose (FPG, mmol/L) / 2]

TyG-body mass index (TyG-BMI): TyG × BMI (kg/m^2^)

TyG-waist-to-height ratio (TyG-WHtR): TyG × (waist circumference (cm) / height (cm)) (24).

TyG-Chinese visceral adiposity index (TyG-CVAI): TyG × CVAI

The CVAI was calculated using a validated, sex-specific formula where lipid parameters are in mmol/L (25):

For males: CVAI = −267.93 + 0.68 × Age (years) + 0.03 × BMI (kg/m^2^) + 4.00 × Waist Circumference (cm) + 22.00 × log10(Triglycerides, mmol/L) – 16.32 × HDL-C (mmol/L)(25).

For females: CVAI = −187.32 + 1.71 × Age (years) + 4.23 × BMI (kg/m^2^) + 1.12 × Waist Circumference (cm) + 39.76 × log_10_(Triglycerides, mmol/L) – 11.66 × HDL-C (mmol/L) (25).

### 2.4 Covariates

Covariates adjusted for in regression models included: age, gender, smoking status (current yes/no), alcohol consumption (never, occasional, frequent), physical activity level (none, < 3 times/week, ≥3 times/week), education level (basic, intermediate, advanced), hypertension status (based on documented history), systolic and diastolic blood pressure, hemoglobin (HGB), alanine aminotransferase (ALT), and estimated glomerular filtration rate (eGFR, calculated using the CKD-EPI 2021 equation) (26). Because HDL-C is already incorporated into the CVAI formula (and thus into TyG-CVAI), it was not included as a separate covariate to avoid overadjustment. We acknowledge that medication history (e.g., glucose-lowering drugs, statins, antihypertensives) and diabetes duration were not available in the EHR and could not be adjusted for; this limitation is addressed in the Discussion.

### 2.5 Statistical analysis

All analyses were performed using R software (version 4.4.0). A two-sided *P*-value < 0.05 was considered statistically significant.

#### 2.5.1 Descriptive statistics

Baseline characteristics of the study population were summarized overall and stratified by CKM stage (2, 3, 4). Continuous variables were presented as mean ± standard deviation (if normally distributed) or median (interquartile range) and compared using ANOVA or the Kruskal-Wallis test. Categorical variables were presented as counts (percentages) and compared using the Chi-square test.

#### 2.5.2 Correlation analysis

The monotonic relationships between the four IR indices and the ordinal CKM stage were assessed using Spearman's rank correlation. A correlation matrix was visualized using a heatmap. A scatter plot depicting the distribution of TyG-CVAI across CKM stages was also generated.

#### 2.5.3 Multivariable regression analysis

Ordinal Logistic Regression: To evaluate the independent association between each IR index and the likelihood of being in a more advanced CKM stage, proportional odds logistic regression models were fitted with CKM stage (2/3/4) as the dependent variable. The proportional odds assumption was tested using the Brant test; no violation was detected (*P* > 0.05 for all models). Results were expressed as adjusted odds ratios (OR) and 95% confidence intervals (CI) per one-standard-deviation increase in each index.

Multiple Linear Regression: to complement the ordinal analysis, multiple linear regression models were constructed with TyG-CVAI as the dependent variable and CKM stage (entered as an ordinal variable) as the primary predictor. This approach assumes a linear trend across CKM stages (i.e., the difference in TyG-CVAI between Stage 2 and Stage 3 is equal to that between Stage 3 and Stage 4). Although this assumption may not fully hold, we used it as a simplified sensitivity analysis to complement the ordinal logistic regression. Three sequentially adjusted models were built:

Model 1: Age + Gender

Model 2: Model 1 + Smoking status + Drinking status + Physical activity + Education level

Model 3: Model 2 + Hypertension status + SBP + DBP + HGB + ALT + eGFR

Note on collinearity: because the CVAI formula already includes age as a component, adjusting for age separately in regression models could introduce over adjustment. However, we retained age as a covariate to assess the independent effect of TyG-CVAI beyond chronological age. Variance inflation factor (VIF) was calculated for all models; VIF values for age and TyG-CVAI were below 2.5, indicating no problematic collinearity.

#### 2.5.4 Subgroup and interaction analyses

Stratified analyses were performed to examine the consistency of the association between TyG-CVAI (as the primary exposure) and CKM stage across predefined subgroups: gender (male/female), age (< 60/≥60 years), and hypertension status (yes/no). The significance of effect modification was tested by introducing a multiplicative interaction term (e.g., TyG-CVAI × subgroup variable) into the fully adjusted ordinal logistic regression model (Model 3). Results from the subgroup-specific models were presented in a forest plot.

#### 2.5.5 Diagnostic performance evaluation

The discriminatory power of each IR index for identifying late-stage CKM (Stages 3–4) was assessed using receiver operating characteristic (ROC) curve analysis. The area under the curve (AUC) was calculated, and pairwise comparisons of AUCs were conducted using DeLong's test. For each index, the optimal cutoff value was determined by maximizing the Youden index (sensitivity + specificity – 1). Corresponding sensitivity, specificity, and positive predictive value (PPV) were reported.

## 3 Results

### 3.1 Baseline characteristics of the study population

A total of 5,609 adults with type 2 diabetes were included in this cross-sectional analysis, comprising 2,922 (52.1%) in CKM Stage 2, 2,017 (36.0%) in Stage 3, and 670 (11.9%) in Stage 4. The baseline characteristics of the overall population and stratified by CKM stages (2, 3, and 4) are presented in Table 1.

**Table 1:** Baseline Characteristics by CKM Stage.

Variable	Overall *N* = 5,609^1^	CKM stage 2 *N* = 2,922^1^	CKM stage 3 *N* = 2,017^1^	CKM stage 4 *N* = 670^1^	*p*-value^2^
**Age**	62.05 (11.74)	58.99 (12.39)	64.14 (9.82)	69.08 (9.57)	< 0.001
Education					
Basic	3,315 (59.1%)	1,793 (61.4%)	1,100 (54.5%)	422 (63.0%)	< 0.001
Intermediate	1,548 (27.6%)	732 (25.1%)	634 (31.4%)	182 (27.2%)	
Advanced	746 (13.3%)	397 (13.6%)	283 (14.0%)	66 (9.9%)	
Physical					
No exercise	1,666 (29.7%)	938 (32.1%)	562 (27.9%)	166 (24.8%)	< 0.001
< 3 times/week	728 (13.0%)	384 (13.1%)	261 (12.9%)	83 (12.4%)	
≥3 times/week	3,215 (57.3%)	1,600 (54.8%)	1,194 (59.2%)	421 (62.8%)	
Drinking					
Never	4,136 (73.7%)	2,532 (86.7%)	1,134 (56.2%)	470 (70.1%)	< 0.001
Occasionally	959 (17.1%)	291 (10.0%)	549 (27.2%)	119 (17.8%)	
Frequently	514 (9.2%)	99 (3.4%)	334 (16.6%)	81 (12.1%)	
Smoking					
Non-smoker	4,617 (82.3%)	2,741 (93.8%)	1,318 (65.3%)	558 (83.3%)	< 0.001
Smoker	992 (17.7%)	181 (6.2%)	699 (34.7%)	112 (16.7%)	
Hypertension	1,174 (20.9%)	520 (17.8%)	417 (20.7%)	237 (35.4%)	< 0.001
Heart_disease	396 (7.1%)	0 (0.0%)	0 (0.0%)	396 (59.1%)	< 0.001
Cerebrovascular	275 (4.9%)	0 (0.0%)	0 (0.0%)	275 (41.0%)	< 0.001
Fatty_liver	1,313 (23.4%)	589 (20.2%)	500 (24.8%)	224 (33.4%)	< 0.001
BMI	24.84 (3.31)	24.51 (3.38)	24.99 (3.21)	25.04 (3.58)	0.007
HGB	139.66 (17.94)	137.08 (16.81)	143.79 (18.36)	138.45 (19.12)	< 0.001
WBC	6.55 (1.75)	6.40 (1.73)	6.73 (1.76)	6.63 (1.77)	< 0.001
PLT	233.95 (63.85)	240.02 (63.81)	228.08 (60.81)	225.14 (70.07)	< 0.001
FPG	8.22 (2.87)	8.03 (2.72)	8.44 (3.02)	8.41 (3.01)	< 0.001
HbA1c	7.65 (1.84)	7.53 (1.79)	7.79 (1.93)	7.76 (1.75)	< 0.001
ALT	25.94 (20.85)	25.75 (19.16)	26.07 (19.74)	26.34 (29.45)	0.125
AST	24.07 (15.86)	24.02 (16.00)	23.69 (14.37)	25.42 (19.11)	0.092
TBIL	13.34 (6.40)	13.28 (6.06)	13.38 (6.46)	13.48 (7.51)	0.892
Cr	78.69 (45.45)	66.61 (17.85)	93.80 (61.07)	85.85 (57.50)	< 0.001
BUN	6.12 (2.94)	5.65 (2.43)	6.68 (3.52)	6.54 (2.64)	< 0.001
TC	4.96 (1.25)	4.97 (1.18)	5.12 (1.29)	4.45 (1.30)	< 0.001
TG	2.03 (1.87)	1.93 (1.56)	2.26 (2.32)	1.77 (1.46)	< 0.001
LDL	3.04 (1.01)	3.05 (0.98)	3.18 (1.03)	2.61 (1.00)	< 0.001
HDL	1.27 (0.35)	1.33 (0.36)	1.20 (0.32)	1.23 (0.35)	< 0.001
uACR	134.09 (585.06)	77.80 (352.12)	194.80 (584.13)	196.80 (1,127.33)	< 0.001
SBP	134.01 (16.86)	130.86 (16.22)	137.44 (16.71)	137.43 (17.37)	< 0.001
DBP	81.54 (10.36)	80.31 (10.03)	83.53 (10.39)	80.93 (10.76)	< 0.001
eGFR	85.62 (21.38)	91.54 (18.01)	79.89 (23.35)	77.06 (21.14)	< 0.001
TyG	9.23 (0.74)	9.10 (0.67)	9.27 (0.75)	9.43 (0.80)	< 0.001
TyG_BMI	229.81 (38.86)	223.45 (37.58)	232.10 (38.53)	236.57 (42.02)	< 0.001
TyG_WHtR	5.03 (0.70)	4.88 (0.68)	5.07 (0.69)	5.23 (0.73)	0.003
CVAI	116.69 (36.14)	110.30 (35.70)	122.18 (35.97)	128.04 (32.96)	< 0.001
TyG_CVAI	1,086.87 (374.79)	1,022.73 (364.26)	1,148.63 (382.68)	1,180.34 (344.40)	< 0.001

^1^Mean (SD); n (%).

^2^Kruskal-Wallis rank sum test; Pearson's Chi-squared test.

Significant differences were observed across CKM stages for nearly all examined parameters. Participants with more advanced CKM stages were notably older, with mean age increasing stepwise from 59.0 years in Stage 2 to 69.1 years in Stage 4 (*P* < 0.001). Distinct patterns were observed in lifestyle factors: the proportion of never-smokers was highest in Stage 2 (93.8%), dropped markedly in Stage 3 (65.3%), and was intermediate in Stage 4 (83.3%) (*P* < 0.001). A similar non-linear trend across stages was noted for alcohol consumption.

As expected, the prevalence of key comorbidities increased with CKM severity. Hypertension rose from 17.8% (Stage 2) to 35.4% (Stage 4), and fatty liver disease from 20.2 to 33.4% (both *P* for trend < 0.001). A history of clinical cardiovascular disease (heart disease or cerebrovascular disease) was present exclusively in the Stage 4 group (59.1 and 41.0%, respectively), consistent with the AHA staging definition.

Metabolic and renal profiles showed marked deterioration across advancing stages. Levels of fasting plasma glucose, HbA1c, systolic and diastolic blood pressure, serum creatinine, blood urea nitrogen, and urinary albumin-to-creatinine ratio were all significantly higher, while estimated glomerular filtration rate (eGFR) and HDL-C levels were lower in advanced stages (all *P* < 0.001). A distinct pattern was observed for lipids: triglycerides (TG) peaked in Stage 3, whereas total cholesterol (TC) and LDL-C were highest in Stage 3 but lowest in Stage 4.

Critically, the four insulin resistance indices of primary interest all exhibited statistically significant and stepwise increases across CKM stages (all *P* ≤ 0.003). The mean TyG increased from 9.10 in Stage 2 to 9.43 in Stage 4. Similarly, TyG-BMI rose from 223.45 to 236.57, and TyG-WHtR from 4.88 to 5.23. The composite index TyG-CVAI demonstrated a clear graded increase, with mean values of 1022.73 in Stage 2, 1148.63 in Stage 3, and 1180.34 in Stage 4 (*P* < 0.001).

### 3.2 Correlation between insulin resistance indices and CKM stage

Spearman rank correlation analysis revealed significant positive monotonic relationships between all four evaluated insulin resistance indices and the ordinal CKM stage (all *P* < 0.001), as detailed in Table 2. The strength of correlation varied notably among the indices.

**Table 2:** Correlation analysis between TyG indices and CKM stage.

TyG index	ρ	95% CI	*P*-value	Effect size	Direction
TyG	0.135	[0.107, 0.160]	< 0.001	Small	Positive
TyG_BMI	0.115	[0.088, 0.142]	< 0.001	Small	Positive
TyG_WHtR	0.154	[0.128, 0.183]	< 0.001	Small	Positive
TyG_CVAI	0.326	[0.302, 0.350]	< 0.001	Medium	Positive

Interpretation: effect size classification: negligible (|ρ| < 0.1), Small (0.1 ≤ |ρ| < 0.3), Medium (0.3 ≤ |ρ| < 0.5), Large (|ρ| ≥ 0.5).

The conventional TyG index and its derivative combining body mass index (TyG-BMI) demonstrated statistically significant but weak positive correlations with CKM stage (ρ = 0.135 and ρ = 0.115, respectively). Incorporating waist-to-height ratio (TyG-WHtR) yielded a slightly stronger, yet still small, correlation (ρ = 0.154).

**Figure 1:**
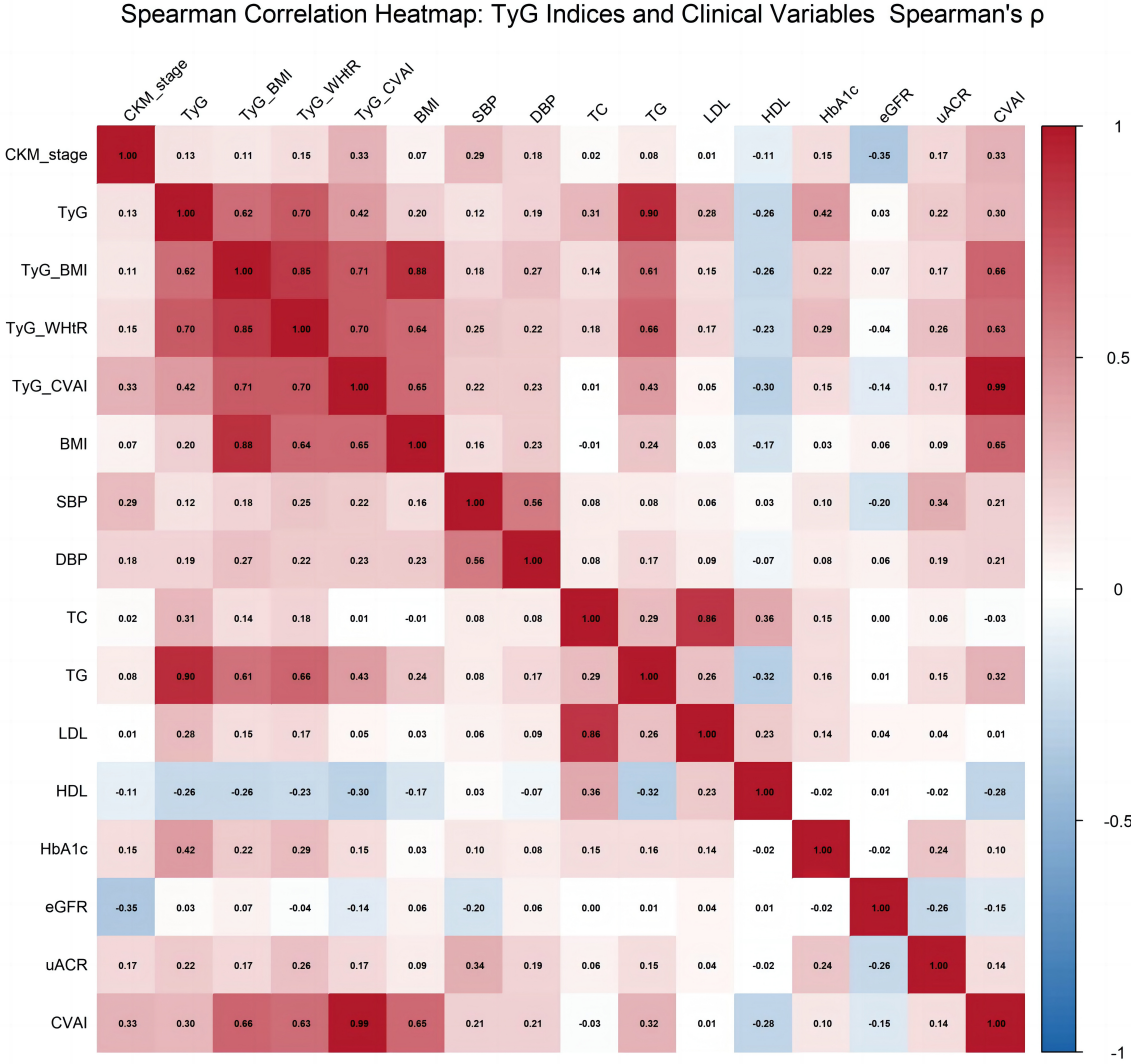
Spearman correlation heatmap: TyG indices and clinical variables (including TyG_CVAI).

Notably, the TyG-CVAI index exhibited the strongest association with CKM stage, with a correlation coefficient of ρ = 0.326 (95% CI: 0.302–0.350), which is classified as a medium effect size. This correlation was substantially stronger than those observed for the other three indices. The correlation heatmap (Figure 1) visually underscores this pattern, highlighting TyG-CVAI as the index most strongly associated with CKM severity. Furthermore, the scatter plot of TyG-CVAI across CKM stages (Supplementary Figure 1) illustrates a clear upward trend in median TyG-CVAI values with each advancing disease stage, visually corroborating the significant positive correlation identified in the quantitative analysis.

### 3.3 Independent association of TyG-CVAI with CKM stage: multivariable regression analyses

To assess the independent relationship between TyG-CVAI and CKM stage, we performed both ordinal logistic regression and multiple linear regression analyses with sequential adjustment for potential confounders.

**Figure 2:**
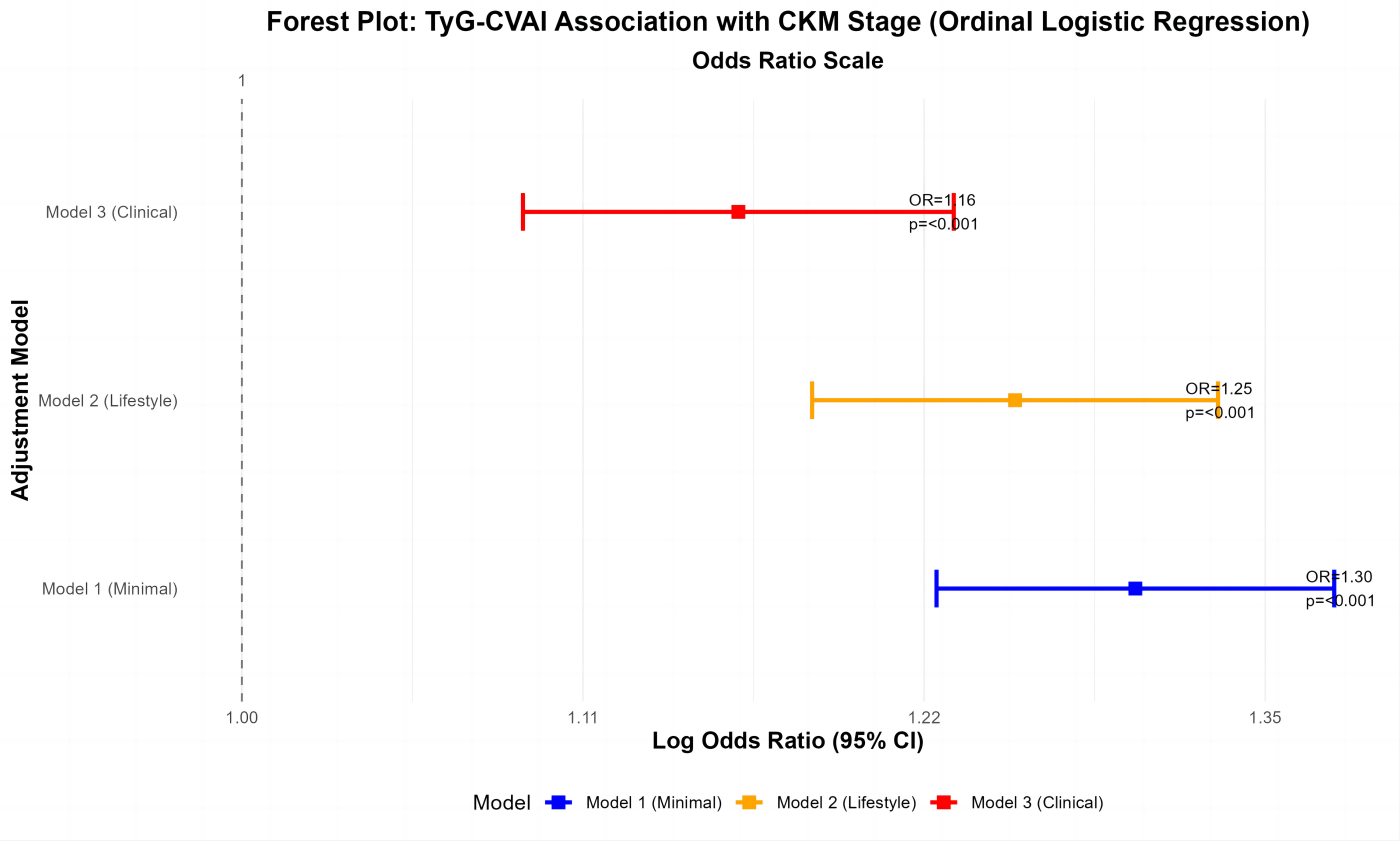
Forest plot: TyG-CVAI association with CKM stage (ordinal logistic regression) odds ratio scale.

In the ordinal logistic regression analysis, where CKM stage (2/3/4) was the dependent variable, a one-standard-deviation increase in TyG-CVAI was consistently associated with significantly higher odds of being in a more advanced CKM stage across all models (Figure 2). After minimal adjustment for age and gender (Model 1), the adjusted odds ratio (OR) was 1.38 (95% CI: 1.29–1.47; *P* < 0.001). This association remained robust after further adjustment for lifestyle factors including smoking, drinking, physical activity, and education level (Model 2: OR = 1.36, 95% CI: 1.28–1.45; *P* < 0.001). Most importantly, in the fully adjusted model that additionally included hypertension status, blood pressure, hemoglobin, ALT, and eGFR (Model 3), the association, though attenuated, remained highly statistically significant (OR = 1.20, 95% CI: 1.11–1.29; *P* < 0.001).

**Figure 3:**
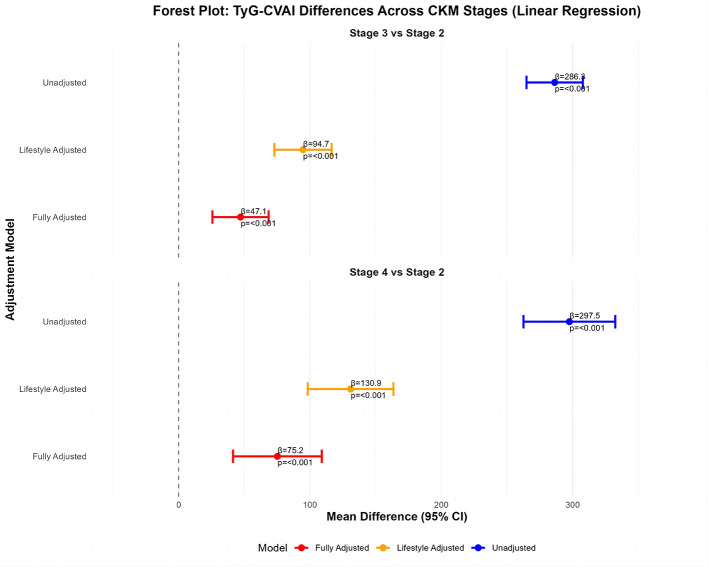
Forest plot: TyG-CVAI differences across CKM stages (linear regression) stage 3 vs. stage 2.

To complement this finding, multiple linear regression was conducted with TyG-CVAI as the dependent variable and CKM stage as the predictor (Figure 3). In the unadjusted model, TyG-CVAI levels were significantly higher in both Stage 3 (β = +286.3 units, 95% CI: 264.8–307.7) and Stage 4 (β = +297.5 units, 95% CI: 262.5–332.4) compared to Stage 2 (both *P* < 0.001). After adjusting for lifestyle factors (Model 2), the differences narrowed but remained substantial and significant (Stage 3 vs. 2: β = +94.7 units; Stage 4 vs. 2: β = +130.9 units). In the final model with full clinical adjustment (Model 3), a significant, graded increase in TyG-CVAI persisted across CKM stages. Specifically, TyG-CVAI was on average 47.1 units higher (95% CI: 25.7–68.5) in Stage 3 and 75.2 units higher (95% CI: 41.4–109.0) in Stage 4, compared to Stage 2 (both *P* < 0.001).

In summary, both regression approaches demonstrated a significant, independent, and graded positive association between TyG-CVAI and the severity of CKM syndrome, even after comprehensive adjustment for a wide range of demographic, lifestyle, and clinical confounders.

### 3.4 Subgroup and interaction analyses

**Figure 4:**
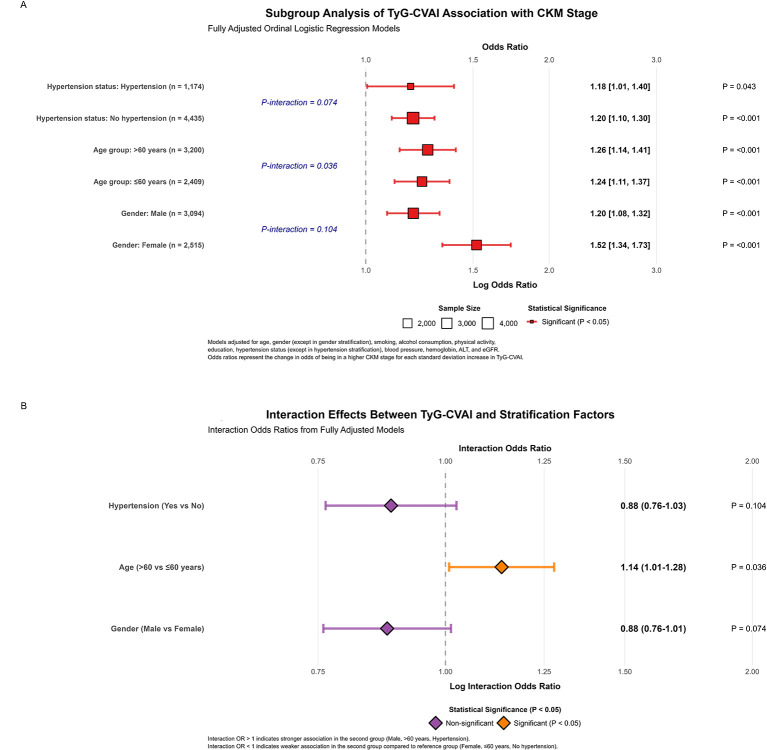
**(A)** Subgroup analysis of TyG-CVAI association with CKM stage fully adjusted ordinal logistic regression models. **(B)** Interaction effects between TyG-CVAI and stratification factors interaction odds ratios from fully adjusted models.

To evaluate the robustness and potential effect modification of the association between TyG-CVAI and CKM stage, we conducted subgroup analyses by gender, age group (< 60 vs. ≥60 years), and hypertension status. The results of the fully adjusted ordinal logistic regression models for each subgroup are presented in a forest plot (Figure 4A).

The association between TyG-CVAI and higher CKM stage was consistently positive and statistically significant across all predefined subgroups (all *P* < 0.05). The point estimates of the odds ratios (ORs) were similar between males (OR = 1.22) and females (OR = 1.18), and between normotensive (OR = 1.20) and hypertensive (OR = 1.19) individuals. A slightly stronger association was observed in participants aged ≥60 years (OR = 1.24) compared to those < 60 years (OR = 1.17).

Formal tests for interaction (Figure 4B) suggested potential effect modification by age group (*P* for interaction = 0.036), with a stronger association observed in participants aged ≥60 years compared to those < 60 years. Given the large sample size, this marginal interaction should be interpreted cautiously and requires validation in independent cohorts. In contrast, the interaction terms for gender (*P* = 0.074) and hypertension status (*P* = 0.104) did not reach the conventional threshold for statistical significance, suggesting that the positive association of TyG-CVAI with CKM stage is largely consistent irrespective of sex or hypertension status.

### 3.5 Diagnostic performance of insulin resistance indices for identifying late-stage CKM

**Figure 5:**
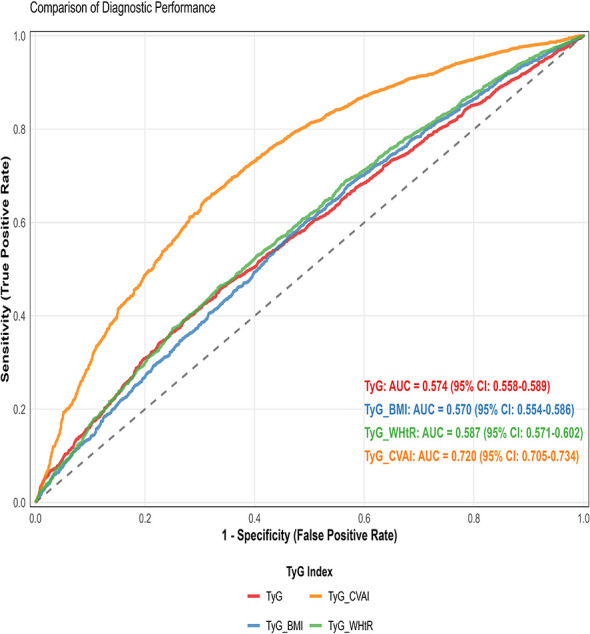
ROC curves for TyG-related indices in predicting CKM stage 3-4 comparision of diagnostic performance.

The discriminatory power of each insulin resistance (IR) index for distinguishing late-stage CKM (Stages 3–4) from Stage 2 was evaluated using receiver operating characteristic (ROC) curve analysis (Figure 5). Table 3 summarizes the corresponding diagnostic performance metrics, including AUC, optimal cut-off, sensitivity, specificity, accuracy, and positive predictive value. TyG-CVAI demonstrated the highest diagnostic accuracy, with an area under the curve (AUC) of 0.720 (95% CI: 0.705–0.734). At its optimal cut-off valueof 1,090.436, determined by maximizing the Youden index, TyG-CVAI achieved a sensitivity of 66.0%, a specificity of 67.9%, an overall accuracy of 66.6%, and a positive predictive value (PPV) of 81.6%.

**Table 3:** Diagnostic performance of TyG-related indicesfor predicting CKM stage 3–4.

Indicator	AUC	95% CI	Best cut-off	Sensitivity	Specificity	Accuracy	PPV
TyG	0.574	[0.558, 0.589]	9.293	46.2%	65.8%	52.4%	74.5%
TyG_BMI	0.570	[0.554, 0.586]	229.371	67.9%	43.0%	60.0%	72.0%
TyG_WHtR	0.587	[0.571, 0.602]	5.122	46.3%	66.4%	52.7%	74.9%
TyG_CVAI	0.720	[0.705, 0.734]	1090.436	66.0%	67.9%	66.6%	81.6%

AUC, Area under ROC curve; PPV, Positive predictive value; Best cut-off determined by Youden's index. The reference group is CKM stage 2, and the case group is CKM stages 3–4.

In contrast, the other three indices showed significantly lower and more modest discriminatory power. The AUCs for TyG, TyG-BMI, and TyG-WHtR were 0.574, 0.570, and 0.587, respectively. Their performance characteristics varied: TyG and TyG-WHtR exhibited higher specificity (65.8 and 66.4%, respectively) but lower sensitivity (46.2 and 46.3%), whereas TyG-BMI showed higher sensitivity (67.9%) but lower specificity (43.0%).

Pairwise comparisons of the AUCs using DeLong's test (Table 4) confirmed that TyG-CVAI significantly outperformed all other indices (all *P* < 0.001). The AUC difference between TyG-CVAI and the second-best performer, TyG-WHtR, was 0.133. Furthermore, the AUC of TyG-WHtR was also statistically superior to that of TyG-BMI (*P* < 0.001) and marginally superior to that of TyG (*P* = 0.046). No significant difference was found between the AUCs of TyG and TyG-BMI (*P* = 0.646).

**Table 4:** Pairwise comparison of AUCs using delong's test.

Comparison	AUC DIFFERENCE	*Z*-statistic	*P*-value
TyG vs. TyG_BMI	0.003	0.459	0.646
TyG vs. TyG_WHtR	−0.013	−1.998	0.046
TyG vs. TyG_CVAI	−0.146	−17.470	< 0.001
TyG_BMI vs. TyG_WHtR	−0.016	−3.548	< 0.001
TyG_BMI vs. TyG_CVAI	−0.149	−25.704	< 0.001
TyG_WHtR vs. TyG_CVAI	−0.133	−22.090	< 0.001

DeLong test was used to compare the areas under ROC curves (AUCs) between different TyG indices. A significant p-value (P < 0.05) indicates statistically significant difference in diagnostic performance.

In summary, the composite index TyG-CVAI provided substantially superior discriminatory ability for identifying late-stage CKM compared to the traditional TyG-based indices, achieving the highest overall diagnostic accuracy in this population with type 2 diabetes. Of note, an AUC of 0.720 indicates moderate discrimination; the clinical utility of TyG-CVAI should be interpreted in the context of its intended use as a simple screening tool rather than a definitive diagnostic test.

## 4 Discussion

In this large, real-world cohort of 5,609 adults with type 2 diabetes, TyG-CVAI demonstrated the strongest and most consistent association with CKM severity compared with three alternative TyG-derived insulin-resistance surrogates (TyG, TyG-BMI, TyG-WHtR). TyG-CVAI showed the largest Spearman correlation with ordered CKM stage, remained independently associated with higher stage after comprehensive covariate adjustment, and yielded substantially superior discrimination for late-stage CKM (AUC = 0.720) vs. the other indices. The association was broadly consistent across sex and hypertension strata and was modestly stronger in participants aged ≥60 years, a pattern that aligns with prior cohort evidence suggesting age-dependent amplification of TyG-based risk signals. This study provides the first direct evidence that among patients with established diabetes, a visceral-adiposity-weighted TyG composite holds greater value for cross-sectional risk identification compared to simpler TyG-based indices within the CKM framework. We also observed non-linear trends in smoking and alcohol consumption across CKM stages. The proportion of current smokers increased from 6.2% in Stage 2 to 34.7% in Stage 3, then decreased to 16.7% in Stage 4. Similarly, the proportion of current drinkers (occasional or frequent) rose from 13.4% in Stage 2 to 43.8% in Stage 3, then fell to 29.9% in Stage 4. These patterns may reflect behavioral changes related to disease progression (e.g., lifestyle modifications after a cardiovascular event) or detection bias, though causal interpretation is precluded by the cross-sectional design.

Our results concord with prior CHARLS-based and national cohort analyses showing that TyG-derived composites that incorporate anthropometric or visceral-fat components improve prediction of cardiovascular and cardiometabolic outcomes. Li et al. reported that TyG-BMI was associated with incident CVD across CKM stages 0–3 with a linear dose-response and identified age as an effect modifier, which mirrors our observation of stronger associations in older participants (18). Similarly, a recent head-to-head comparison of nine TyG-related indices in a CHARLS sample found TyG-CVAI to achieve the highest AUC for cardiometabolic multimorbidity, supporting the generalizability of TyG-CVAI's superior performance in Chinese middle-aged and older adults (21). Those prospective cohort data and our cross-sectional diabetes-specific analysis together suggest that integrating visceral-adiposity metrics into TyG composites yields meaningful incremental prognostic information in CKM-relevant populations.

The superiority of TyG-CVAI may be explained by its integration of complementary pathophysiologic axes into a single metric. TyG, as the product of fasting triglycerides and fasting glucose, has been validated as a simple surrogate for insulin resistance and is associated with cardiovascular outcomes (27). CVAI is a validated, sex-specific estimator of visceral adiposity that includes age, BMI, waist circumference, TG, and HDL-C, and has shown good predictive performance for visceral fat burden and cardiometabolic risk in several Chinese population cohorts (28, 29). The product of the two (TyG × CVAI) amplifies signals of dysglycemia, atherogenic dyslipidemia, visceral fat burden, and age-related risk accumulation, thereby more comprehensively reflecting pathways that promote cardiorenal organ damage. Each of these axes contributes to endothelial dysfunction, systemic inflammation, and renal microvascular injury—processes implicated in both CVD and CKD—so an index that synthesizes them would be expected to align closely with multi-organ disease severity (30, 31). This integrative rationale is further supported by mediation analyses from NHANES showing that TyG-related metrics partially mediate the link between accelerated biological aging and late-stage CKM, with central-adiposity-weighted indices (e.g., TyG-WHtR and TyG-BMI) explaining a substantial portion of the association (32).

Visceral adiposity itself is a plausible driver of multimorbidity: it is metabolically active, can promote insulin resistance, and secretes proinflammatory cytokines that in turn facilitate atherogenesis and organ fibrosis (33). Therefore, indices that better approximate visceral fat burden (such as CVAI and WHtR) provide incremental information compared with overall obesity measures represented solely by BMI (34), which may explain why TyG-CVAI outperformed TyG-BMI in our diabetic cohort. Moreover, because CVAI includes age in its formula, this may partly account for the stronger TyG-CVAI effect observed in older participants: age both increases cumulative exposure to cardiometabolic insults and alters body-fat distribution, so an index that explicitly incorporates age can better capture age-dependent risk gradients (35).

Clinically, TyG-CVAI is attractive because it is calculable from routine laboratory and anthropometric data commonly available in primary-care and community settings. In patients with type 2 diabetes—who occupy a central, high-risk position on the CKM continuum—TyG-CVAI could be used as an EHR-friendly screening tool to flag individuals for enhanced monitoring and intensified cardiovascular and renal evaluation (e.g., albuminuria testing, more frequent eGFR monitoring, or targeted cardiac assessment). Within the AHA CKM framework, which emphasizes stage-appropriate prevention, TyG-CVAI may help identify patients who are already at a more advanced CKM stage and thereby inform decisions about lifestyle interventions and pharmacotherapies with proven cardiorenal benefit (e.g., SGLT2 inhibitors and GLP-1 receptor agonists). However, the moderate AUC (0.720) indicates that TyG-CVAI should be used in conjunction with other clinical factors rather than as a standalone test. Implementation research will be required to define optimal action thresholds and to evaluate whether TyG-CVAI-guided care pathways improve clinical outcomes. External validation in diverse populations and healthcare settings is essential before clinical adoption.

Our subgroup and interaction analyses revealed that age significantly modified the association between TyG-CVAI and CKM stage, with a stronger association observed in participants aged ≥60 years. This finding aligns with prior studies showing age-dependent amplification of TyG-related risk signals, likely due to cumulative exposure to cardiometabolic insults and age-related changes in body fat distribution (35). Notably, the interaction was marginal (*P* = 0.036) and should be interpreted cautiously given the large sample size; validation in independent cohorts is needed. No significant effect modification was observed for sex or hypertension status, suggesting that TyG-CVAI performs consistently across these subgroups. These results support the potential utility of TyG-CVAI as a broadly applicable screening tool in diverse T2D populations.

Despite these strengths, several limitations warrant consideration. First, the cross-sectional design prevents causal inference about whether elevated TyG-CVAI precedes CKM progression. Longitudinal validation in prospective diabetes cohorts is necessary to establish temporal ordering and utility for incident cardiorenal events. Second, CKM staging in our study relied on pragmatic EHR proxies (e.g., Framingham risk estimates and diagnostic codes), which may misclassify subclinical disease. Direct assessment of subclinical CVD (e.g., coronary calcium score, echocardiography) was not available. Nevertheless, this approach enhances the feasibility of large-scale, real-world studies and our operational definitions were aligned with the AHA advisory (1). Third, the cohort was drawn from a single regional health system in China, so external validation across diverse ethnic and healthcare settings is needed before broad generalization. Fourth, we lacked detailed data on medication history (e.g., glucose-lowering drugs, statins, SGLT2 inhibitors, GLP-1 receptor agonists, antihypertensive agents) and diabetes duration. These unmeasured variables could influence TyG-CVAI values and CKM stage, and their absence may lead to residual confounding. We fully acknowledge this as a major limitation, as these medications have proven cardiorenal benefits and may independently affect CKM stage, potentially biasing our estimates. We urge future studies to incorporate detailed medication and disease duration data to better control for confounding. Fifth, residual confounding by factors such as diet, socioeconomic status, and other unmeasured comorbidities cannot be fully excluded despite multivariable adjustment. Finally, the modest AUC of 0.720 suggests that while TyG-CVAI outperforms simpler indices, its clinical utility as a standalone screening tool is moderate; it should be integrated with established risk factors rather than replace them.

Future research should prioritize prospective evaluation of TyG-CVAI for incident cardiorenal events and test whether interventions that reduce TyG-CVAI—through weight loss, lipid-lowering, or glucose-lowering therapies that preferentially reduce visceral fat—translate into slower CKM progression or fewer clinical events. Comparative effectiveness studies are also needed to determine clinically actionable thresholds and to evaluate the incremental value of TyG-CVAI when embedded in multivariable risk models or decision support tools. Future studies should also employ net reclassification improvement (NRI) and decision curve analysis (DCA) to better quantify the clinical utility of TyG-CVAI beyond traditional risk factors. Mechanistic investigations linking longitudinal changes in TyG-CVAI to biomarkers of inflammation, fibrosis, and endothelial dysfunction would further clarify causal pathways and may identify intermediate targets for intervention.

## 5 Conclusions

This comparative study demonstrates that in adults with type 2 diabetes, TyG-CVAI—a composite index unifying metabolic and visceral adiposity signals—exhibits a stronger association with CKM staging and provides improved discrimination of late-stage CKM compared to simpler TyG-based indices. These results support the further prospective validation of TyG-CVAI as an accessible and practical biomarker that may assist in risk stratification, though its moderate discriminatory accuracy (AUC = 0.720) suggests it should be used in conjunction with established clinical factors rather than as a standalone test.

## Data Availability

The data analyzed in this study is subject to the following licenses/restrictions: The datasets presented in this article are not publicly available due to privacy and ethical restrictions. The data originate from electronic health records of community health centers and contain sensitive patient information. Access to the data was granted by the Medical Ethics Committee of Longhua District People's Hospital of Shenzhen for the purpose of this retrospective study under a license agreement, with all data processed in a de-identified format. Requests for data access should be directed to the corresponding author and will be subject to approval by the relevant ethics and data governance committees, contingent upon a formal data sharing agreement. Requests to access these datasets should be directed to Corresponding author contact: 13418850411@139.com.
