# Supplementary figures and images for: The Triglyceride-Glucose-Chinese Visceral Adiposity Index (TyG-CVAI) outperforms other insulin resistance indices in its association with cardiovascular-kidney-metabolic syndrome severity in type 2 diabetes: a comparative study
Source: Front Nutr. 2026 May 8;13:1817071. doi: 10.3389/fnut.2026.1817071 (PMC13194601; doi:10.3389/fnut.2026.1817071)

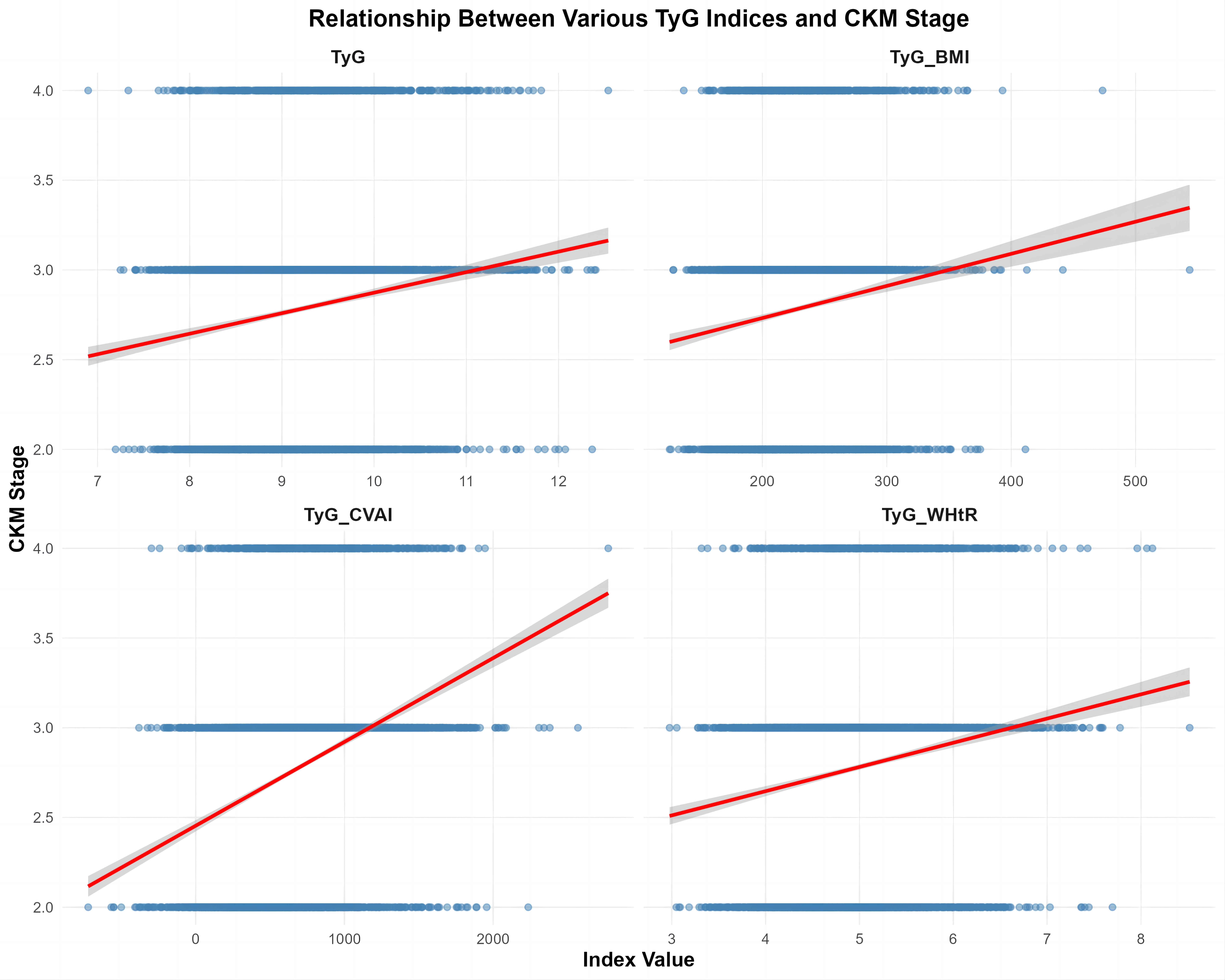

Supplement: Supplementary file 1 [file Image_1.jpeg]
